# Novel scoring system combined with a virtual reality technique for the preoperative evaluation of the stone-free status after flexible ureteroscopy: the H.L.P.E.S. score

**DOI:** 10.1186/s12894-022-01108-2

**Published:** 2022-10-08

**Authors:** Jianglin Gu, Shengjun Luo, Li Jiang, Daixing Hu, Guozhi Zhao, Wei Tang

**Affiliations:** Department of Urology, The First Affiliated Hospital of Chongqing Medical University, Chongqing, China

**Keywords:** Flexible ureteroscopy, Renal calculi, H.L.P.E.S score, Stone-free rate, Urolithiasis, Ureteroscopy lithotripsy

## Abstract

**Objective:**

The original S.O.L.V.E. scoring system was modified using virtual reality technology, and a new H.L.P.E.S scoring system was constructed to improve the accuracy of predicting the stone-free rate after flexible ureteroscopy.

**Methods:**

We retrospectively analyzed clinical and virtual reality data of 150 patients with renal calculi who underwent flexible ureteroscopy at the First Affiliated Hospital of Chongqing Medical University, Chongqing, China, from September 2019 to January 2022. Factors affecting the stone-free rate were evaluated in univariate and multiple logical regression analyses. Factors were divided by cut-off value under the receiver-operating characteristic curve and scored accordingly to a well-known international scoring system. Area under the curve predicted the stone-free rate. The accuracy and superiority of the stone-free rate after flexible ureterorenoscopy was compared between this scoring system and the S.O.L.V.E, R.I.R.S, T.O.HO, and RUSS scores.

**Results:**

Multiple logistic regression showed that the stone surface area, renal pelvis volume, and length of the calyces funnel were correlated with stone-free rate (*P* < 0.01, *P* = 0.021, *P* = 0.019, respectively). The H.L.P.E.S. score included stone surface area (1–2 points), renal pelvis volume (1–2 points), length of calyces funnel (1–2 points), pelvic calyceal height (1–2 points), and essence of stone (1–2 points). The area under the receiver-operating characteristic curve of H.L.P.E.S. score was 0.927, which was higher than the S.O.L.V.E., R.I.R.S., T.O.HO, and RUSS scores.

**Conclusion:**

H.L.P.E.S. scoring can effectively predict the stone-free rate after flexible ureteroscopy for renal calculi and is superior to other scoring systems.

## Introduction

Urolithiasis is one of the most common urological diseases. The epidemiological data of Europe and America show that 5–10% of the population experiences urolithiasis at least once in their lifetime, and the new incidence rate is 100 − 400/100,000 [1] every year. The incidence in China is about 6.5%, with the south having a higher incidence than the north does [1]. In recent years, flexible ureterorenoscopy (FURS) has been accepted by most patients because of its high efficiency, minimal invasiveness, and safety. A first-line treatment of FURS of the upper urinary tract calculi < 2cm has been recommended by the European Association of Urology, American Urological Association, and Canadian Urological Association [2]. Although percutaneous nephrolithotomy remains the gold standard for the treatment of complex renal calculi with a diameter of > 2cm, the development of laser technology, auxiliary equipment, and consumables has made the use of FURS for the treatment of complex renal calculi possible and it is now recommended by the major guidelines as second-line treatment. However, because of the low stone-free rate (SFR), multiple operations are required. Xu et al. reported [3] that FURS and holmium laser were effective even in patients with a renal calculus burden > 40mm. Even if cases of relatively large stones, this trend is likely to continue. A different SFR might occur in cases in which different treatment methods are used for the same stone size. Determining the treatment method to achieve the highest SFR is a major problem for clinicians. The determinants of SFR after FURS are not uniform and are influenced by multiple factors, including stone characteristics and renal anatomy [4, 5]. Currently, there are many reports in the literature on this issue [4–9]. Previous scoring systems including STONE [10], CROES Calculation Chart [11], and Guy’s stone score, [12] among others, are applicable only to percutaneous nephrolithotomy, and there is a lack of evidence for a predictive model for SFR after FURS [13]. In the early stage, we built the SOLVE scoring system based on computed tomography (CT) scan and three-dimensional (3D) reconstruction, but some errors have been noted in the measurement accuracy and method, and its clinical value is limited. With the recent wide application of the virtual reality (VR) technique in the surgical field, the measurement accuracy of many variables has been greatly improved. Thus, we used the VR technique to modify the previously established S.O.L.V.E. score and to create a novel scoring system, referred to as the H.L.P.E.S. score, and compare it with other established scoring systems.

## Methods

### Data collection and ethical statements

This study was approved by the Ethics Committee of the First Affiliated Hospital of Chongqing Medical University. We retrospectively collected clinical data and data related to VR measurements from 150 patients with renal calculi treated with FURS in our hospital between September 2019 and January 2022. No preoperative CT of urography (CTU) or VR image processing and renal anatomical abnormalities were excluded.

### Methods of measurement and operation

**Fig. 1:**
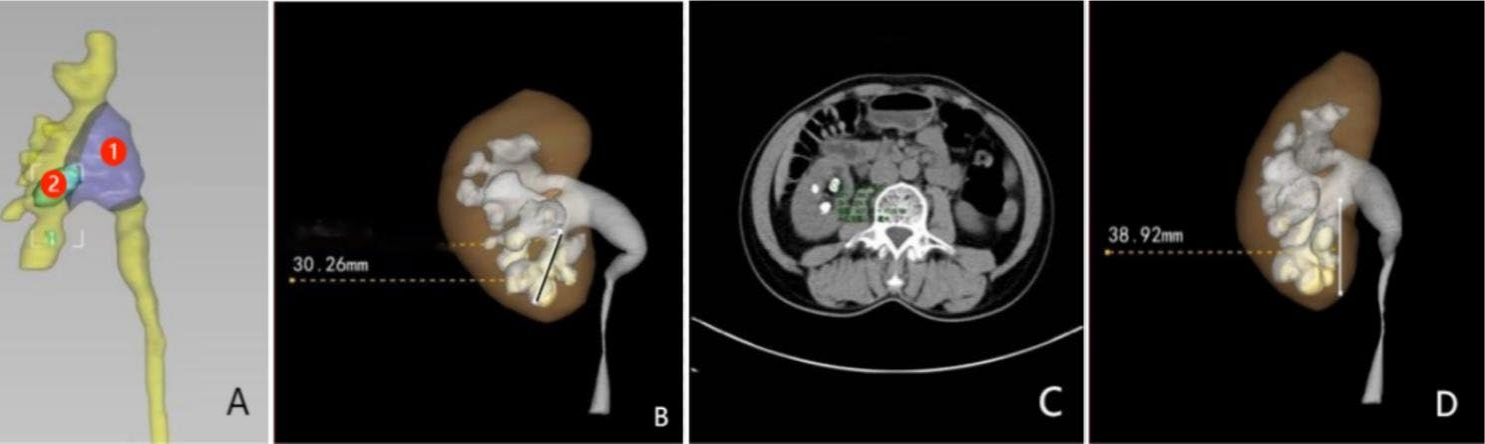
(**A**) VR imaging and measurement. 1: Renal pelvis volume (one end is the junction of the renal pelvis and ureter; the other end is the portion of the renal pelvis except for renal calyx). 2: Stone surface area. (**B**) Length of the calyces funnel (distance between the farthest point of the lower calyx where the calculus is located and the middle point of the lower lip of the renal pelvis). (**C**) Essence of stone (average CT value of the calculus). (**D**) Pelvic calyceal height (vertical distance between the horizontal line of the lowest point of the lower calyces where the calculus is located and the highest point of the lower lip)

CTU was obtained and VR techniques were used to measure the stone surface area, renal pelvis volume, length of the calyces funnel, essence of stone, and pelvic calyceal height. All measurements were obtained using a holographic 3D image reconstruction system (version 1.0, Ruisheng Medical Technology Co., Ltd., Chongqing High-tech Industrial Development Zone). Figure1 shows the specific measurement method. We excluded patients with ureteral or renal anomalies or calyceal diverticula and those with unavailable data.

A total of 150 patients with renal calculi underwent FURS under general anesthesia. First, we used F8/9.8 rigid ureteroscopy to examine the ureteropelvic junction with a 0.038-inch zebra guidewire. An F12/14 ureteroscopic sheath was inserted into the ureteropelvic junction along the guidewire, and ureteroscopic examination of the renal pelvis and calyces was performed. We inserted a 200-µm holmium laser fiber through the operation channel. The laser energy and frequency were set to 1.2J/15–20Hz. The stone was crushed to < 4mm by the holmium laser. The larger stone was removed from the body using a sleeve basket. After the operation, an indwelling F16 catheter and F6 ureter stent were placed.

## Definition of stone-free status

A patient was defined as stone-free when no residual stone or residual fragments < 4mm were detected on kidney, ureter, and bladder X-ray imaging carried out 1 month after surgery [8].

### Statistical analysis

The measurement data of normal distribution or of approximate normal distribution are expressed as the mean ± standard deviation, and the measurement data of partial distribution are expressed in terms of median (minimum to maximum). The relationships of evaluation factors with stone-free status were analyzed using chi-square test or a two-tailed unpaired Student’s t-test. All possible predictors were analyzed by univariate analysis, and statistically significant predictors were included in the multivariate analysis. We used a logistic regression model to analyze the relevant factors affecting the SFR, and we calculated the cutoff values of each factor by drawing the receiver-operating characteristic (ROC) curve; the value was used as the boundary value of each factor. We calculated the value of the area under the curve (AUC) of the HLPES scoring system to predict the SFR. We compared the AUC of the H.L.P.E.S. with that of the S.O.L.V.E, R.I.R.S, T.O.HO., and RUSS scores. P < 0.05 was considered statistically significant. We used SPSS software (version 25.0; IBM Corporation, Armonk, NY, USA) for statistical analyses.

## Results

We included a total of 150 patients (102 men and 48 women) with a mean age of 49.6 ± 12.0 years (range, 23–77 years). Patients were divided according to postoperative stone-free status. The stone-free group included 120 cases: 79 men and 41 women, mean age 49.27 ± 11.99 years, 34 cases of previous stone surgery, median body mass index 24.3kg/m^2^ (range, 16.5–31.0kg/m^2^), 25 cases had an indwelling ureteral stent before the operation, 61 cases of calculi located on the left side, and 59 cases of calculi located on the right side. The stone-residual group included 30 cases: 23 men and 7 women, average age 51.00 ± 12.23 years, 10 cases a history of stone operation, median body mass index 24.9kg/m^2^ (range, 18.8–33.1kg/m^2^), three cases had an indwelling ureteral stent before the operation, 19 cases had calculi located on the left side, and 11 cases had calculi located on the right side. Table1 shows the specific data.

The total SFR after FURS was 80% (120/150). Table1 shows the associations between stone-free status and stone characteristics. Significant differences were observed between the stone-free group and stone-residual group in stone surface area, renal pelvis volume, length of the calyces funnel, and essence of stone but not for pelvic calyceal height.

**Table 1:** Comparison of patient characteristics according to postoperative stone-free status

Variables	stone-free group(n = 120)	stone- residual group(n = 30)	P value
Age, years	49.27 ± 11.99	51.00 ± 12.23	0.482†
Gender, n (%)			
Male	79(65.8%)	23(76.7%)	0.255*
Female	41(34.2%)	7(23.3%)	
BMI, kg/m^2^	24.29 ± 3.11	25.24 ± 3.37	0.143†
Affected side(n, %)			
Left	61(50.8%)	19(63.3%)	0.220*
Right	59(49.2%)	11(36.7%)	
Preoperative stent (n, %)			
Yes	25(20.8%)	3(10.0%)	0.173*
No	95(79.2%)	27(90.0%)	
Prior treatment(n, %)			
Yes	34(28.3%)	10(66.7%)	0.591*
No	86(71.7%)	20(33.3%)	
Stone surface area,mm^3^ [14]	513.9 ± 406.1	1844.3 ± 1179.4	**<0.001**
Renal pelvis volume,mm^3^ [14]	2240.0 ± 2019.7	5472.2 ± 7092.6	**0.019**
Visible number of calyces	1.96 ± 1.6	2.27 ± 2.5	0.553†
Essence of stone,HU	973.3 ± 360.4	1240.8 ± 411.4	**<0.001**
Length of calyces funnel,mm	20.9 ± 6.1	28.7 ± 6.1	**<0.001**
Pelvic calyceal height,mm	22.4 ± 7.7	26.0 ± 7.7	0.052†
Obstruction by S.O.L.V.E.,mm	16.5 ± 6.0	15.0 ± 5.0	0.216†
Operation time, min	29.3 ± 15.4	54.2 ± 30.83	**<0.001**

Abbreviation: BMI, body mass index

*Pearson’s chi-square test

†No significant difference between the same superscripts. Bold font indicates statistical significance (P < 0.05)

In the multivariate regression analysis, we included the four variables found to be statistically significant from the univariate analysis and the approximately statistically significant variable of pelvic calyceal height. Although the *P* value of pelvic calyceal height was > 0.05, its odds ratio was > 1, which allowed us to consider it a risk factor for residual stones. The results showed that stone surface area, renal pelvis volume, and length of the calyces funnel were correlated with SFR (*P* < 0.01, *P* = 0.021, and *P* = 0.019, respectively), whereas essence of stone and pelvic calyceal height were not (*P* > 0.05; Table2).

**Table 2:** Uni- and multivariate logistic regression analysis findings for stone-free rate

	Univariate analysis		Multivariate analysis	
	**OR(95%CI)**	**P value**	**OR(95%CI)**	**P value**
Stone surface area,mm^3^	1.00(1.00–1.00)	<0.001	1.00(1.00 ~ 1.00)	0.002
Renal pelvis volume,mm^3^	1.00(1.00–1.00)	0.001	1.00(1.00 ~ 1.00)	0.021
Length of calyces funnel,mm	1.20(1.11–1.31)	<0.001	1.18(1.03 ~ 1.35)	0.019
Pelvic calyceal height,mm	1.06(1.00-1.13)	0.057	1.01(0.91 ~ 1.11)	0.902
Essence of stone,HU	1.00(1.00–1.00)	0.001	1.00(1.00–1.00)	0.200

### Establishment of the H.L.P.E.S. scoring system

**Fig. 2:**
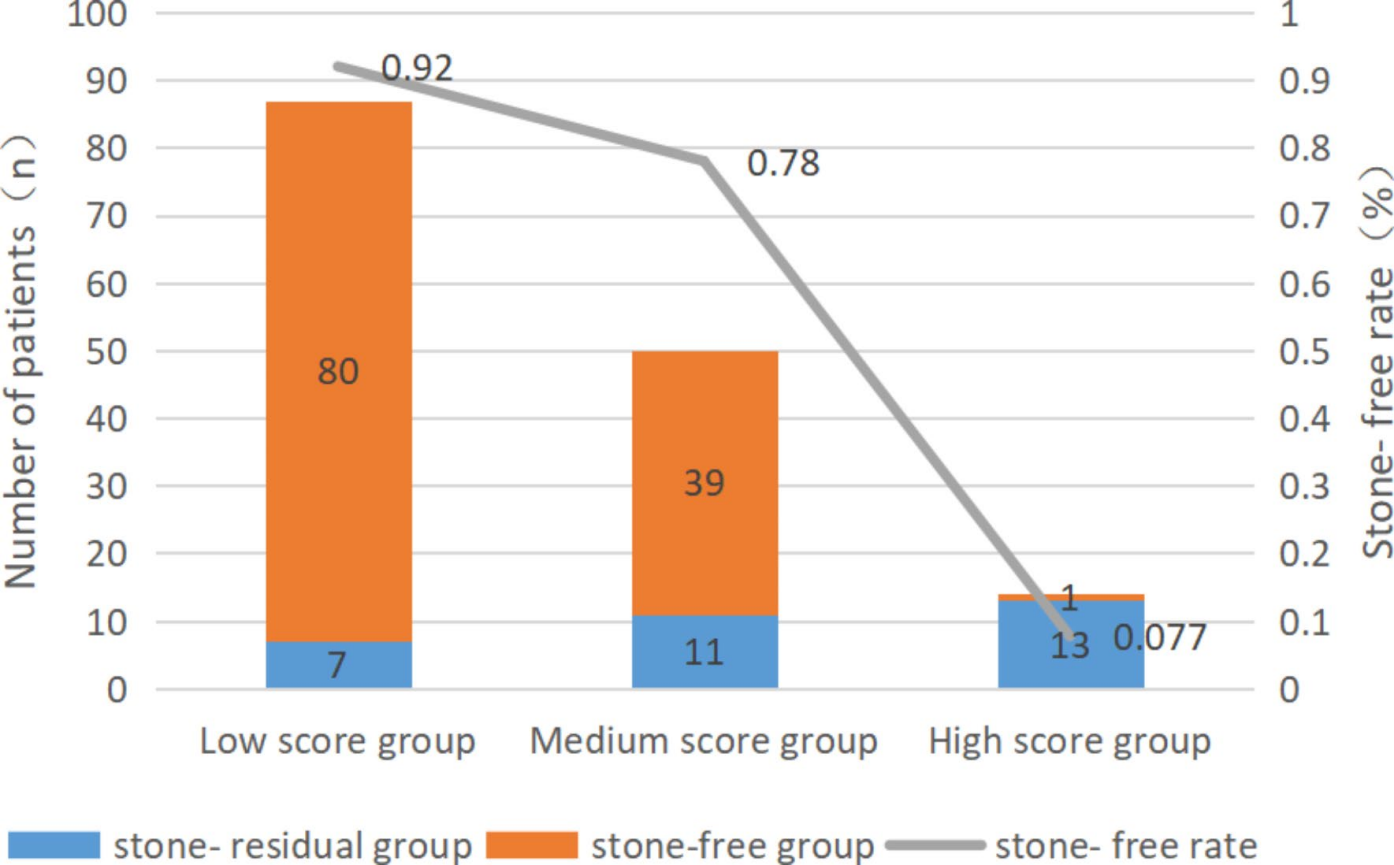
Stone-free rate after flexible ureteroscopy according to H.L.P.E.S. scoring

The revised scoring system includes the stone surface area, essence of stone, renal pelvis volume, length of calyces funnel, and pelvic calyceal height. For each factor, the cut-off values are taken as the boundary values. Each variable is assigned a value in reference to the previous literature and the original S.O.L.V.E score [4–9]: (S)tone surface area (1–2 points); renal (P)elvis volume (1–2 points); (L)ength of the calyces funnel (1–2 points), for which the pyelolithiasis score should be 0; pelvic calyceal (H)eight (1–2 points), for which pyelolithiasis is recorded as 0 points; and CT value as the evaluation standard of the (E)ssence of stone (1–2 points). The specific scores are shown in Table3. From the results of the references [10–17] and statistical analysis according to the SFR of different scores, we divided scores into low (3–6 points), middle (7–8 points), and high (9–10 points). The total score ranged from 3 to 10 points. The average H.L.P.E.S. score was 6.4 points, among which 87 cases had a low score, 50 had a medium score, and 13 had a high score. The operative times were 27.0 ± 19.0, 42 ± 20.0, and 53 ± 22.0min for the low-, medium-, and high-score groups, respectively, with postoperative SFR values of 92% (80/87), 78% (39/50), and 7.7% (1/13), respectively, all of which were statistically significant (*P* < 0.01). Figure2 shows the H.L.P.E.S. scores of the different patient groups with SFR and trends.

**Table 3:** Description of the HLPES system

	Score	
	**1 pt**	**2 pt**
Pelvic calyceal (H)eight,mm	≤ 21	>21
(L)ength of calyces funnel,mm	≤ 25	>25
Renal (P)elvis volume,mm^3^	≤ 2214	>2214
(E)ssence of stone,HU	≤ 1425	>1425
(S)tone surface area,mm^3^	≤ 969	>969

**Fig. 3:**
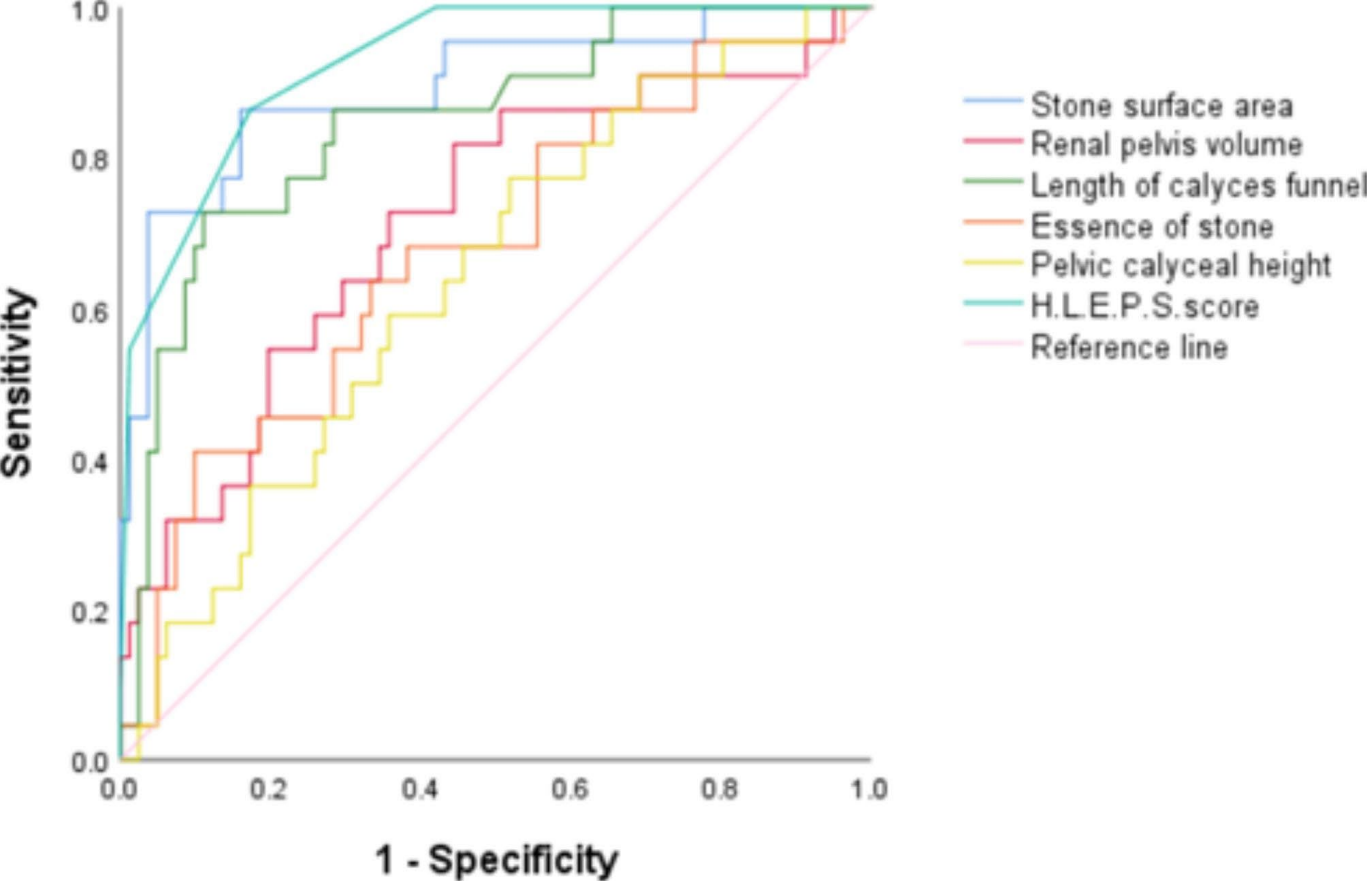
ROC on the inpact to the stone-free rate from H.L.P.E.S Scoring and its parameters

We constructed the new H.L.P.E.S. score by incorporating the five variables mentioned above. Figure3 shows the ROC curve of the score and its indices affecting SFR after FURS. The area under the ROC of the H.L.P.E.S. score was 0.927, which was higher than any of the variables in the score (Table4).

**Table 4:** Area under the ROC curve of the HLPES score and its parameters

	AUC	95%CI	P value
H.L.P.E.S.score	0.927	0.874 ~ 0.980	<0.01
(S)tone surface area,mm^3^	0.893	0.809 ~ 0.978	<0.01
Renal (P)elvis volume,mm^3^	0.715	0.589 ~ 0.842	0.002
(L)ength of calyces funnel,mm	0.848	0.756 ~ 0.940	<0.01
Pelvic calyceal (H)eight,mm	0.639	0.514 ~ 0.763	0.047
(E)ssence of stone,HU	0.676	0.546 ~ 0.806	0.012

**Fig. 4:**
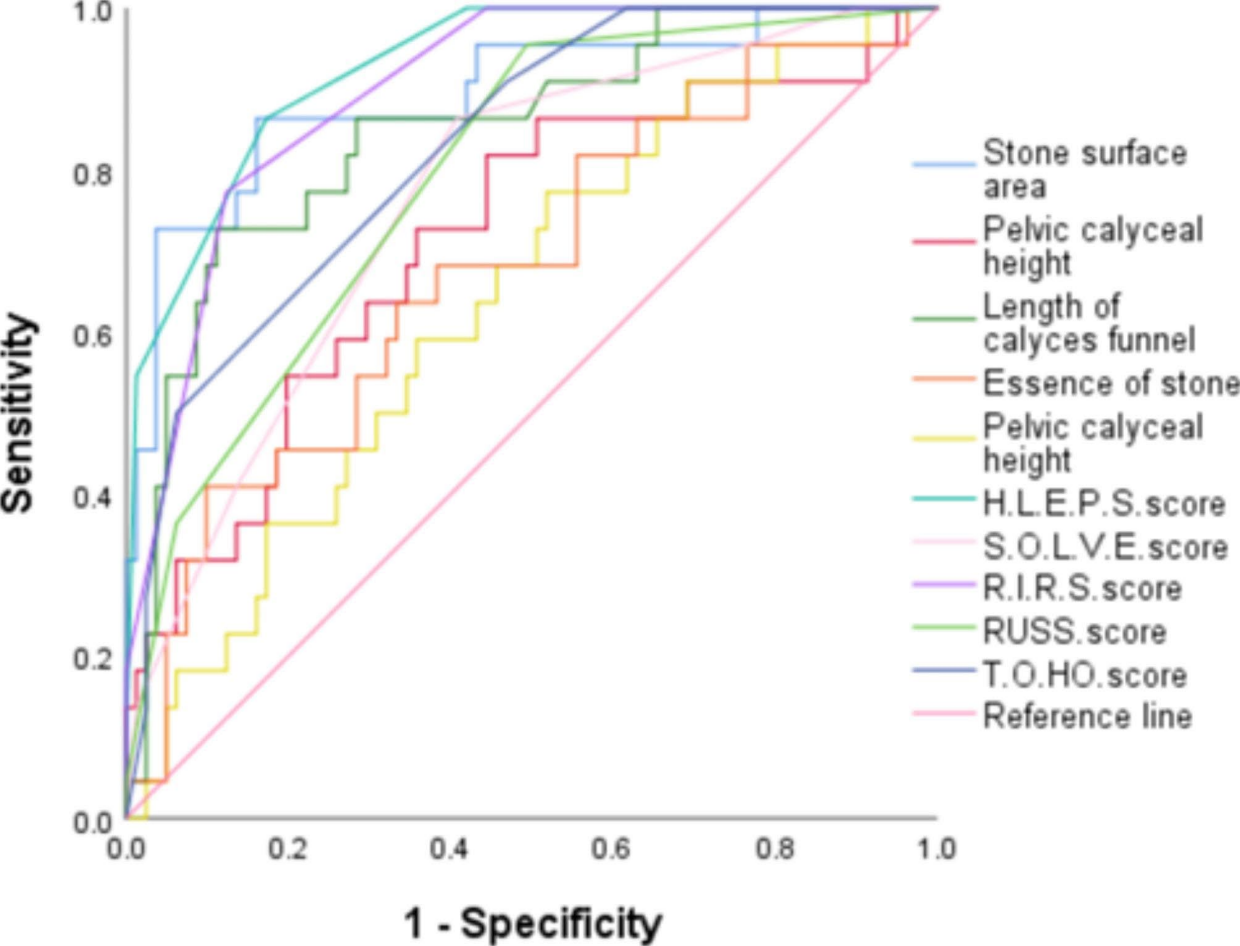
ROC curves for predicting stone-free status with five scoring systems

To further verify its accuracy and predictive value, we compared the H.L.P.E.S. score with the existing R.I.R.S., RUSS., S.O.L.V.E., and T.O.HO. scores, as shown in Fig.4. Table5 shows the cut-off value, sensitivity, and specificity of each scoring system.

**Table 5:** Cutoff, sensitivity, specificity, and AUC values of the HLPES, RIRS, TOHO, RUSS, and SOLVE scoring systems for predicting treatment failure

	Cut-off	Sensitivity(%)	Specificity(%)	AUC (95% CI)	P value
H.L.P.E.S. score	7.5	86.4	82.7	0.927(0.874 ~ 0.980)	<0.01
R.I.R.S.score	7.5	77.3	87.7	0.899(0.836 ~ 0.962)	<0.01
RUSS score	0.5	95.5	50.1	0.792(0.694 ~ 0.890)	<0.01
T.O.HO.score	7.5	90.9	53.1	0.827(0.738 ~ 0.917)	<0.01
S.O.L.V.E.score	8.6	86.4	59.3	0.764(0.658 ~ 0.871)	<0.01

## Discussion

The technology used in medical optical equipment, laser equipment, and auxiliary material equipment has developed rapidly, and the treatment of renal calculi has changed greatly. FURS has many advantages over other methods, including higher SFR, less trauma, and quicker recovery. FURS has quickly become an effective minimally invasive method for treating renal calculi. SFR is the ultimate goal of all surgeons, but FURS removes kidney stones through the body’s natural lumen; thus, many factors affect SFR after FURS, including urinary system anatomy and stone characteristics. Because the preoperative point of departure of stones with identical sizes is often different based on the choice of surgical treatment, differences occur in SFR, which affects the surgical results. Patients would undoubtedly benefit from an evaluation system that can effectively predict the preoperative stone removal rate and guide the prediction of surgical methods. Previously, some errors were found in the accuracy and measurement methods of the S.O.L.V.E scoring system, limiting its clinical value. With the recent widespread application of VR in the surgical field, we can accurately evaluate the effect of FURS using the H.L.P.E.S. score and VR before the operation, which will assist clinicians in determining optimal treatment. Previous scoring variables remain an important factor for predicting SFR, but the accuracy of variable measurement is limited by imaging techniques; thus, the value of the prediction is affected. The new scoring system retains the previous stone surface area, length of calyces funnel, and essence of stone and adds renal pelvis volume and pelvic calyceal height. In addition, VR technology application makes the measurement of each variable more accurate and will undoubtedly greatly improve the prediction accuracy.

Essence of stone leads to variations in operation time. Ito et al. [18] found a significant correlation between the CT value of the calculus and lithotripsy efficiency but no significant effect on postoperative SFR. In this study, stone density was assigned two points, and the boundary value was 1425 HU. The average CT value of the stone-free group was lower than that of the group with stone residue.

The stone surface area is an important factor in the absence of residual stones [19] and was assigned two points, with a boundary value of 969 mm^3^ [2]. The study by Yamashita et al. [4] found that for every 1-cm [14] increase in stones, the postoperative residual stone rate increased 1.8 times (OR = 1.791, 95% CI 1.345–2.653), and the risk of reoperation increased. In this study, stone size differed significantly (OR = 1.002, *P* = 0.002) between the non-residual group and the residual group, and as the score increased, there was a significant difference in postoperative SFR (*P* < 0.01) between the groups [20].

The calyces funnel length is an important factor affecting SFR after FURS. A renal calyx infundibulum that is too long will result in a large distance from the renal calyx stone to the renal pelvis, leading to residual stone. Geavlete et al. [21] found that the SFR was 88.2% for calyx < 30mm and 61.1% for calyx > 30mm. Multivariate analysis showed that calyx length was correlated with SFR. In this study, scores were assigned based on a boundary of 25mm (OR = 1.180, *P* = 0.019). The longer the infundibular part of the calyx, the more difficult it is to treat calculi with FURS, and the more difficult it is to remove calculi.

Renal pelvis volume is another important factor affecting SFR after FURS. Severe hydronephrosis leads to enlargement of the kidney volume and affects the ureteroscopy flexibility. It is easy to miss in the process of searching for stones [21], which leads to a decrease in the stone removal rate. Meanwhile, the very large renal collecting system leads to stone displacement in the process of lithotripsy, which increases the difficulty of lithotripsy and operation time. This study found that the original S.O.L.V.E. score represented the degree of obstruction in the pyelo-calyceal separation, which was not statistically significant in this study. To the best of our knowledge, there have been no published reports of renal pelvis volume, and this is the first study to demonstrate the extent of obstruction in terms of pelvic volume. We creatively used the volume of the pelvis to indicate the degree of obstruction. Two points were assigned to a boundary of 2214 mm^3^ [14], and the difference was statistically significant (OR = 1.000, *P* = 0.021) between the non-residual group and the residual group.

In this study, we measured pelvic calyceal height, which is the length of the vertical line from the base of the calyx to the junction of the pelvic and calyx. This measurement was assigned two points, with a boundary of 21mm. The mean height of the group without residual stone was lower than that of the group with residual stone, which was nearly significant in the single-factor analysis but had no statistical difference in the correlation analysis. We believe that the greater the height and the deeper the depth of the soft lens into the kidney, the more flexible the soft lens will be. The more difficult the calculus is to drain, the more likely it is that there will be residual calculus. Nevertheless, the new H.L.P.E.S. scoring system, which incorporates the height and density of the pelvic and calyx into the scoring system, has an AUC of 0.927, indicating that it is still valuable for postoperative comprehensive prediction of SFR. However, further study is needed with more cases. In their study, Symes et al. [22] concluded that the height of the renal pelvis and calyx is a predictor of success in extracorporeal shock wave lithotripsy for subrenal calyceal calculi, but the effect of pelvic calyceal height in flexible ureteroscopy has not been reported.

In the correlation analysis of H.L.P.E.S. score variables, the H.L.P.E.S. score for SFR was 0.927, which is higher than for any of the variables in the score. Comparisons were made with the existing R.I.R.S., RUSS., S.O.L.V.E., and T.O.HO. scores. H.L.P.E.S. had an AUC of 0.927, which is higher than any of the other scores. The cut-off value of the H.L.P.ES score was the same as that of the R.I.R.S. score or the T.O.HO. score, but the H.L.P.E.S. score had higher in sensitivity and specificity. The new H.L.P.E.S. score was superior to the previous S.O.L.V.E. score in terms of cut-off value, sensitivity, and specificity. The RUSS score was superior to the cut-off value and sensitivity values of each scoring system, but its specificity and AUC values were significantly lower than the newly established H.L.P.E.S. score. Therefore, we believe that this scoring system is superior to other scoring systems. Of course, this study is was single-center, retrospective study with a relatively small sample size, and the results might include selective bias. Thus, we must expand the sample size and actively carry out multicenter, prospective studies to improve the reliability and practicability of the results.

In conclusion, the H.L.P.E.S. scoring system based on VR technology combined with the revised S.O.L.V.E. score can be used not only to evaluate and more accurately predict the calculus clearance rate after FURS but also to strengthen the communication between doctors and patients using the help of VR technology, so as to guide the choice of operation method and achieve accurate medical treatment. The relevant measurement data used in the H.L.P.E.S. scoring system can be easily obtained, and the score itself is of simple, operable, and has a higher predictive value, making it superior to other scoring systems.

## Data Availability

The datasets generated and analysed during the current study are not publicly available due it contains identifiable patient variables. but are available from the corresponding author on reasonable request.

## Abbreviations

3D: three-dimensional.
AUC: area under the curve.
CT: computed tomography.
MoVIES: Monongahela Valley Independent Elders Survey.
PRISMA: Preferred Reporting Items for Systematic Reviews and Meta-Analysis.
CTU: computed tomography of urography.
FURS: flexible ureterorenoscopy.
ROC: receiver-operating characteristic.
VR: virtual reality.
HU: Hounsfield unit.
HLPES: (S)tone surface area, renal (P)elvis volume, (L)ength of the calyces funnel, pelvic calyceal (H)eight, (E)ssence of stone.
RIRS: (R)enal stone density, (I)nferior pole stone, (R)enal infundibular length, (S)tone burden.
TOHO: (T)allness, (O) ccupied lesion and (HO) unsfield units evaluation.
SOLVE: (S)tone surface area, (O)bstruction, (L)ength of the calyces funnel, (V)isible number of calyces, (E)ssence of stone.
RUSS: Resorlu-Unsal Stone Score.
